# Endothelial function assessment by flow-mediated dilation of the brachial artery in acute kidney injury and chronic kidney disease

**DOI:** 10.22088/cjim.14.4.668

**Published:** 2023

**Authors:** Elham Ramezanzadeh, Sima Fallah Arzpeyma, Azin Vakilpour, Mohammadsadegh Abedi, Soheil Hassanipour

**Affiliations:** 1 Razi Clinic Research Development Center, Gilan University of Medical Sciences, Rasht, Iran; 2 Cardiovascular Diseases Research Center, Department of Cardiology, Heshmat Hospital, School of Medicine, Gilan University of Medical Sciences, Rasht, Iran

**Keywords:** Acute kidney injury, Chronic kidney disease, Endothelium, Endothelial dysfunction, Flow-mediated dilation.

## Abstract

**Background:**

Endothelial dysfunction has a significant role in the pathogenesis of cardiovascular events in patients with kidney dysfunction. The present study aimed to compare the level of endothelial dysfunction in patients with chronic kidney disease (CKD) and acute kidney injury (AKI) by brachial artery flow-mediated dilation (FMD) technique. Also, we sought to find whether this non-invasive technique may assist in accurately distinguishing the acute or chronic nature of kidney failure in patients presenting with uremia for the first time.

**Methods:**

Demographic and medical characteristics, and laboratory and renal ultrasonography data of the patients with AKI and CKD were collected and compared with a control group. Brachial artery FMD was measured using a Toshiba aplio 300 device with a 7.5 MHz linear probe.

**Results:**

In a total of 175 patients with a mean (SD) age of 55.96(15.54) years, FMD% was significantly lower in the CKD and AKI patients compared to the control group (Mean±SD: 16.28%±10.52%), 16.28 %±4.35%, and 24.24±5.71, respectively, p<0.001). Among the different causes of AKI, contrast-induced nephropathy (10.78%±1.75%), volume depletion (14.87%±1.22%), and post-renal AKI (15.96%±1.54%) had the lowest levels of FMD. Also, a significant correlation between FMD and eGFR (r=0.26, *P*<0.001), serum Hb (r=0.18, *P*=0.013), Na (r=0.19, *P*=0.011), BUN (r=-0.21,* P*=0.005) and Cr (r=- 0.13,* P*=0.084) was reported.

**Conclusion:**

Compared to the control group, CKD and AKI patients showed greater levels of endothelial dysfunction as evidenced by lower brachial artery FMD. However, the FMD technique did not appear to be a practical method in differentiating CKD and AKI in patients presenting with uremia for the first time.

Patients with chronic kidney disease (CKD) demonstrate an increased risk of cardiovascular mortality and morbidity even at earlier stages (1). In spite of decades of improved care, their high-risk profile persists, and cardiovascular diseases continue to be the primary cause of death in this population (2). On the other hand, acute kidney disease (AKI), like CKD, has been a public health concern for decades due to its adverse long-term outcomes and high mortality rate (3). AKI is considered a potential factor for developing CKD since a significant number of AKI patients require lifelong dialysis or might experience decreased kidney function after the acute phase of the disease (4-6). 

Besides mutual risk factors between CKD and CVD such as aging, hypertension, diabetes mellitus, hyperlipidemia, and smoking, CKD-specific non-classical risk factors including inflammation, volume overload, anemia, uremic toxins, renin-angiotensin, and sympathetic nervous systems, and oxidative stress highly contribute to CVD progression patients suffering from CKD (7). A vast body of literature has identified endothelial dysfunction as an important pathophysiological mechanism of atherosclerosis, and it is suggested to have a significant role in the pathogenesis of CVD in patients with impaired renal function (8, 9).

 Although many factors, such as inflammation-mediated changes in vascular smooth muscle cell function and structural changes like vascular calcification, can contribute to the development of vascular impairment in CKD, endothelial cells dysfunction, which is primarily measured as impaired vascular dilation, is one of the most common concerns (6). Endothelial dysfunction induces vasoconstriction, inflammation and thrombosis, platelet aggregation, and monocyte adhesion, which all contribute to developing atherosclerosis (10). The mechanisms that lead to endothelial dysfunction in CKD are multidimensional and seem to increase as the disease progresses. Early mechanisms likely involve a reduction in nitric oxide (NO) synthesis and bioavailability due to oxidative stress and endogenous inhibition of NO synthase (11). Because measuring endothelium-derived NO appears to be challenging, vascular endothelial function is usually assessed via indirect methods (12). Endothelial dysfunction can be clinically evaluated by increased blood flow shear. B-mode ultrasound has been widely used for evaluating brachial artery flow-mediated dilation (FMD), which is the most extensively used method to assess endothelial cell function (13).

 FMD is a non-invasive technique that serves as a marker for an underlying atherosclerosis risk. After blocking the brachial artery for 5 minutes, the secretion of nitric oxide is stimulated, leading to dilation of the artery (14, 15). Not only is this technique used to assess the severity and extent of coronary artery disease, but it also provides prognosis information for patients with CVD (13). 

In clinical practice, accurately diagnosing the acute or chronic nature of kidney failure in patients presenting with uremia for the first time is commonly required, particularly in developing regions without a good medical record system. In this regard, differentiating acute from chronic renal failure might be challenging when patients present with uremia and whose kidney function has been unclear for three months earlier (16). Given the importance of endothelial cell function in the progression of atherosclerosis and cardiovascular events in patients with various stages of kidney disease, we aimed to compare the level of endothelial dysfunction measured by flow-mediated dilation (FMD) of the brachial artery in CKD and AKI patients and to find whether brachial artery FMD could be a practical tool in accurately diagnosing the acute or chronic nature of kidney failure in patients presenting with uremia for the first time.

## Methods

**Study Protocol: **The present cross-sectional study was conducted on patients with AKI and CKD referred to an outpatient clinic in Razi Hospital, Iran, in 2020. Patients with AKI and CKD were included and classified into two separate groups of AKI and CKD and were compared with the third group of patients with normal blood Cr level (control group) who did not have any history of kidney disease. Patients with the presence of either kidney damage or decreased kidney function for 3 months, were included in the CKD group irrespective of cause (17). The criteria for including patients in AKI group were as follows:

Increase in serum creatinine of ≥0.3 mg/dL within 48 hours or ≥50% within 7 days OR urine output of <0.5 mL/kg/hour for >6 hours (17). Active smokers, patients with a history of collagen-vascular diseases, and those who had cancer were excluded. Also, in the AKI and the control group, patients with a history of comorbidities such as hypertension and diabetes got excluded from the study. After a complete explanation of the purpose of the study for the participants, along with explaining that the brachial artery ultrasound is a completely safe modality, written informed consent was obtained from the eligible patients. All participants were able to leave the study at any time if they were reluctant to continue. This study was approved by the Gilan University of Medical Sciences Ethics Committee (research code: IR.GUMS.REC.1399.167), and the study protocol was carried out according to the guidelines of the 2013 version of the Helsinki Declaration.

**Data collection and ultrasonographic evaluation: **The entire study population underwent kidney ultrasonography performed by well-trained radiologists. Demographic characteristics, medical history (DM, HTN, CHF, single kidney, history of kidney transplantation), laboratory information, and kidney ultrasonography data were collected from the patients' files. All ultrasound assessments were carried out by the radiologist of the team, blinded to the patients' clinical history. Patients were requested to abstain from caffeine, alcohol, vitamin supplementation consumption, and doing exercise for at least 10 hours before testing. Upon arrival, fasting participants laid down and rested in a supine position for 15 minutes in a temperature-controlled quiet room. Patients' blood pressure was measured using a mercury sphygmomanometer which was fit to the individual arm (80% of the diameter and 2/3 of the arm's length). Brachial artery FMD was measured non-invasively according to the standard protocol (18). In brief, participants laid down in a supine position on an examination bed. A standard blood pressure cuff was placed 3-5 cm above the brachial artery pulse site. FMD measurements were done using a Toshiba applio 300 device with a 7.5 MHz linear probe. The brachial artery was imaged above the antecubital fossa in the longitudinal plane. Following obtaining the baseline image, arterial occlusion was induced by the cuff inflation to 50 mmHg above systolic blood pressure for 5 minutes to induce hyperemia. Digital imaging was acquired 1 minute prior to cuff inflation and immediately after cuff deflation. Finally, the FMD was measured as the percentage of change in diameter over the baseline. 

**Statistical analysis:** Mean (standards deviation), median (range), or frequency (percent) statistics were used to describe the data. The normal distribution of the quantitative data was assessed using the Q-Q plot or Shapiro-Wilk test. To compare the measured variables in the studied groups according to the normality of the distribution of variables and homogeneity of variances, one-way analysis of variance (ANOVA), multiple comparison test (Turkey) or Welch test, and non-parametric tests (Kruskal Wallis and Mann-Whitney test) were used. To compare FMD in terms of quantitative and qualitative variables, Pearson correlation, independent t-test, and ANOVA test were applied. A *p*-value of less than 0.05 was considered significant. All data were analyzed using IBM SPSS 26 Version.

## Results

A total of 175 patients with a mean (SD) age of 55.96(15.54) were studied (range: 14-88), of whom 125 patients were assigned to the CKD group (each stage of CKD=25 patients), 25 to the AKI group and 25 patients were studied as the control subjects. In total, 50.8% of the studied population were females. 

Table 1 represents the demographic and clinical characteristics of the studied patients. In the present investigation, a significant difference in the mean of FMD percentile was found between the studied groups (*p*<0.001). So, FMD in the control group was significantly higher than CKD and AKI patients (mean ± SD: controls: 24.24±5.71, AKI group: 16.28 %± 4.35%, CKD group: 16.28%±10.52%). Also, when comparing every two groups, a significant difference was found in the mean FMD of the control group compared to the AKI (*P*=0.006) and CKD subjects (*P*= 0.001). However, no statistically significant differences regarding the mean of FMD between the CKD and AKI groups were observed (*P*=0.969). According to age subgroup analysis, in the group of patients < 45 years old, the mean of FMD in the control group (n=12, Mean SD: 25.75.17) was significantly higher than AKI (n=4. Mean SD: 19.072.5) and CKD subjects (n=25, Mean: 13.517.18). (p <0.001). Also, in the group of patients older than 45 years, the mean of FMD percentile in the control group (n=13, Mean SD: 22.756.03) was found to be significantly higher than AKI (n=21. Mean SD: 15.744.45) and CKD patients (n=100, MeanSD: 16.9511.10). (p<0.017). In addition, in the comparison of FMD percentile between males and females, no differences were found (P=0.43). 

**Figure1:**
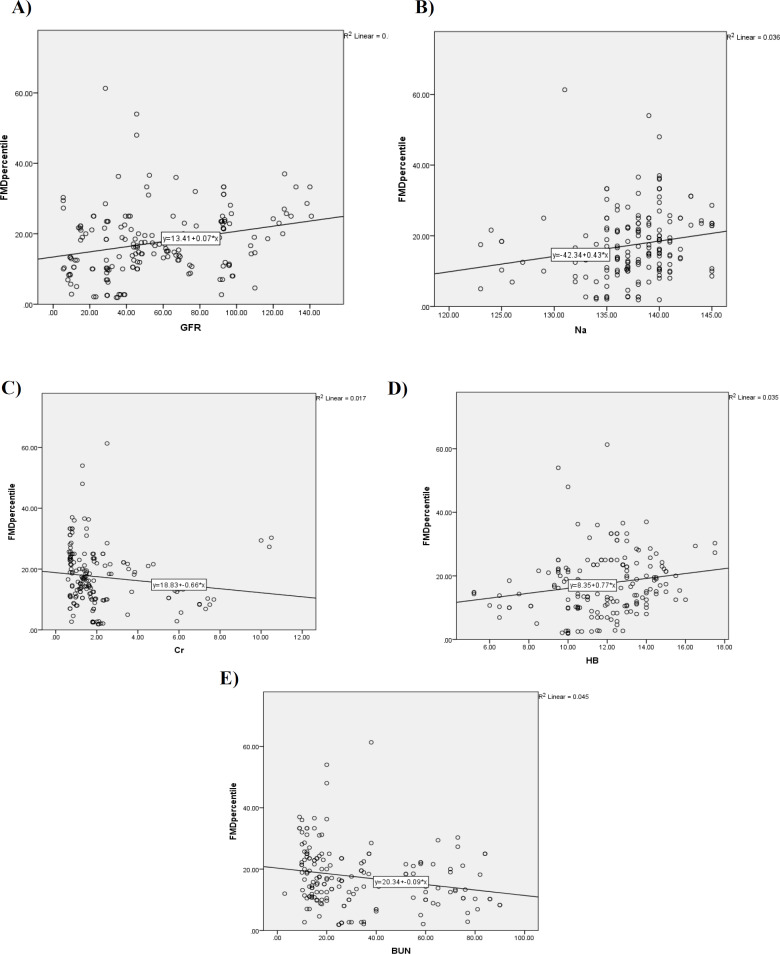
Correlation between brachial artery FMD percentile and laboratory parameters. A: GFR, B: Na, C: Cr, D: Hb, and E: BUN

Figure 1 represents the correlation of FMD with laboratory parameters. Accordingly, a significant positive correlation between serum Hb and sodium (Na) level with FMD was detected (r=0.18, *P*=0.013, r=0.19, *P*=0.011, respectively). Also, serum urea (*P*=0.005, r= -0.21) and creatinine (*P*=0.084, r= - 0.13) showed a weak negative correlation with FMD. Regarding the mean of FMD in stages of CKD, we classified CKD patients into two groups of GFR<30 and GFR≥30. Although FMD level was higher in patients with GFR≥30 (Mean±SD:19.36%±22.20%) compared to the group of patients with GFR lower than 30 (Mean± SD: 14.49%± 10.51%), the difference did not appear to be statistically significant between the two groups (*P*=0.61). Albeit, in the correlation analysis, a significant positive correlation between FMD percentile and eGFR was reported (r=0.26, *p*<0.001).

**Figure 2:**
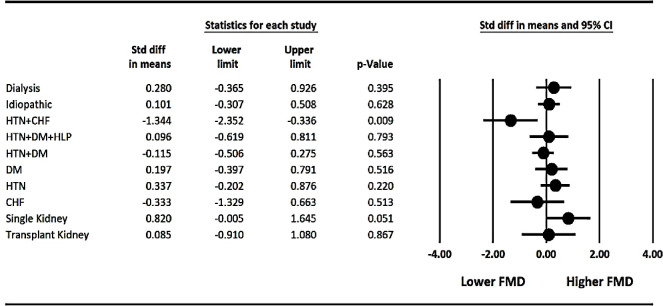
FMD level between different groups of CKD patients with traditional risk factors for CVD and those without CV risk or any comorbidities

Among AKI group, the mean of FMD with respect to the different causes of AKI (post-renal, contrast-induced, glomerulonephritis, rhabdomyolysis, and volume depletion) showed that the lowest level of FMD was seen in contrasted induced nephropathy (FMD percentile mean± SD: 10.78%±1.75%), volume depletion (FMD percentile mean± SD: 14.87%±1.22%), and post-renal AKI (FMD percentile mean± SD: 15.96%±1.54%), respectively. When comparing the FMD level between groups of patients having the traditional risk factors for CVD and those without CV risk or any comorbidities, except for individuals suffering from concurrent hypertension and CHF that had significantly lower FMD level compared with others, no significant differences were detected among those individuals with CV risk factors or comorbidities and those with no known underlying diseases (figure 2). Also, in a binary comparison of the mean FMD between different groups of underlying diseases in CKD patients, a significant FMD mean difference was found in the group of patients with concurrent CHF and hypertension compared to the group suffering from either diabetes or hypertension alone (*P*=0.012, *P*=0.02). 

**Table 1:** Baseline and clinical characteristics of studied patients classified as CKD, AKI, normal control groups

**Characteristics**	**All patients(n=175)**	**CKD(n=125)**	**AKI(n=25)**	**Control(n=25)**	**P-value**
**Age, Mean (SD),y**	55.96±15.53	57.75±16.01	57.44±9.38	45.42±13.94	0.001
**Male sex (%)**	49.2	53.8	40	50	0.570
**eGFR, Mean (SD), ml/min**	56.19±34.69	49.59±31.44	40.35±11.88	104.89±22.32	<0.001
**HB,Mean(SD), (g/dl)**	11.77±2.29	11.90±2.11	9.41±2.27	13.41±1.17	<0.001
**BUN,Mean(SD),( mg/dl)**	31.30±22.96	33.96±24.34	37.12±17.03	12.18±2.12	<0.001
**Cr,Mean(SD), (mg/dl)**	2.02±1.88	2.29±2.09	1.89±0.88	0.78±0.31	0.001
**Na,Mean(SD), (mEq/L)**	137.53±4.21	136.90±4.07	137.44±4.64	140.80±2.82	<0.001
**K,Mean(SD), (mEq/L)**	4.59±2.74	4.72±3.21	4.35±0.37	4.20±0.21	0.605
RK size ,Mean(SD), (cm)*	101.41±17.32	98.23±18.78	105.72±9.75	112.96±6.49	<0.001
LK size ,Mean(SD), (cm)*	103.08±16.65	99.75±17.76	109.96±11.45	113.15±5.81	<0.001
**RK Paranchyme,Mean(SD), (mm)**	11.14±2.54	10.52±2.64	12.32±1.40	13.07±1.19	<0.001
**LK Paranchyme ,Mean(SD), (mm)**	11.50±2.42	10.96±2.48	12.60±1.84	13.15±1.31	<0.001
**FMD (%)**	17.47±9.67	16.28±10.52	16.28±4.35	24.24±5.71	<0.001

All participants were patients referred to an outpatient’s clinic for nephrology visits. e GFR: estimated glomerular filtration rate, HB: hemoglobin, BUN: blood urea nitrogen, Cr: creatinine, Na: sodium, K: potassium, RK: right kidney, LK: left kidney, FMD: flow-mediated dilation.

## Discussion

To the best of our knowledge, this is the first study investigating and comparing endothelial dysfunction measured by FMD in 3 groups of CKD, AKI, and normal kidney function patients to survey whether FMD measurement could be considered as a valuable tool in further differentiation of these prevalent disorders and to predict the CVD prognosis of AKI and CKD patients.

The present study showed that the mean of brachial artery FMD, which is representative of arterial vasodilation impairment, differed significantly between the CKD and AKI patients compared with the controls. So, CKD patients had the lowest mean of FMD percentile, suggesting that patients with CKD had more profound vascular dysfunction compared to the AKI and control groups; however, there was not found any differences in the mean of FMD percentile between the AKI and CKD groups. In the same vein, in a previous investigation, Tal Kopel et al. demonstrated that vascular function was impaired in patients with advanced chronic kidney dysfunction compared with individuals with no overt vascular disease(9). Also, Sharma et al., in a similar investigation, revealed that CKD patients significantly had lower FMD levels in comparison with the control group, and impaired FMD was detected in 26.8% of the patients with CKD, and in line with our findings, they found no difference in the mean of FMD among females vs. males (19).

In a case-control study in 2015, Ito et al. who aimed to determine the usefulness of FMD in CVD prognosis reported that FMD had a significant positive correlation with estimated glomerular filtration rate in diabetic patients with concurrent CHF (20). Previous studies have also revealed abnormal brachial artery FMD in patients with CKD, particularly those with ESRD and those receiving hemodialysis (21-24). FMD measures the brachial artery's ability to respond with endothelial nitric oxide (NO) release when there is reactive hyperemia. Since a healthy endothelium is needed to release NO, reduced FMD is considered a marker of impaired endothelial function. Nevertheless, along with a well-working endothelium, the flow-mediated vasodilatory response requires an arterial reaction to NO, which is an endothelium-independent process. Impairment of nitric oxide reactivity occurs as a result of changes in smooth muscle cell function or structural alterations in the artery (9). 

In this study, the mean of brachial artery FMD in AKI patients was found to be reduced compared to the control group. Significant evidence exists that endothelial dysfunction is important to the etiology of acute renal damage. Also, acute kidney injury is associated with alterations in a vascular tone that contribute to an overall reduction in GFR (25). In this regard, animal studies have shown that ischemia causes changes in endothelial function, which contribute considerably to the overall degree and severity of a kidney damage (25). The present study investigating the mean of FMD concerning the different causes of AKI (post-renal, contrast-induced, glomerulonephritis, rhabdomyolysis, and volume depletion) suggested that FMD might be a useful method in differentiating the causes of acute injury to the kidneys. As such, it was shown that among different reasons, the lowest level of FMD was seen in contrasted induced nephropathy, volume depletion, and post-renal AKI, respectively. Based on the results of some previous studies, contrast media could result in a reduction of cofactors that are involved in NO synthesis, such as tetrahydrobiopterin, or alter substrates, including L-arginine, or intrude with its synthesis via the nuclear factor κB (NFκB), which ultimately prohibits inducible NO synthase mRNA transcription. Some have assumed that this is played by endothelium impairment, which can be attributed to metabolic conditions, which thereafter causes acute kidney failure due to a decreased NO after administration of contrast media (26). 

With regard to the mean of brachial artery FMD in different stages of the CKD severity, in the present study, although a negative correlation between serum Cr and BUN with the percentage of FMD was reported, with increasing CKD severity based on GFR (30 ≤GFR > 30), no significant decrease in the percentage of FMD was observed. Previous studies on FMD evaluation in different grades of CKD showed contrasting results. Similar to our finding, in the study by Thambyrajah et al., there was no significant difference in FMD between lower and upper quartiles of GFR in CKD patients (27). However, other studies revealed that with increasing the severity of CKD, FMD values cut down progressively (28, 19). Furthermore, despite low FMD in the CKD patients, except for patients with concurrent HTN and CHF that had a significantly higher level of endothelial dysfunction based on FMD measurement, there was no significant difference in the mean of FMD percentile between CKD patients with single kidney, transplant kidney and those with traditional CVD risk factors such as HTN, DM, HLP and those without any known comorbidity. This finding suggests that it might be primarily CKD that is responsible for endothelial impairment in patients. Likewise, in a previous investigation, it was reported that in children with CKD -where CVD risk factors have not had the time to occur- reduced FMD has also been observed (19). 

The findings of this study have to be seen in the light of some limitations—first, the relatively small sample size of the study could underestimate the differences between studied groups. Second, the cross-sectional nature of the investigation inhibits inferences about causality. Also, our control subjects’ exclusion criteria for impaired kidney function were based on blood Cr rather than eGFR, and the control group may have included some people with reduced eGFR, which could have led to an underestimation of differences between kidney failure and control groups.

In the present study, CKD and AKI patients were reported to have higher levels of vascular endothelial dysfunction, as evidenced by decreased brachial artery FMD compared to the control group. Despite low FMD in CKD patients, endothelial function was impaired equally in CKD subjects with and without traditional CV risk factors. Finally, although the FMD technique did not appear to be practical in differentiating CKD and AKI in the study subjects, it could be considered a helpful method in differentiating the causes of AKI by reporting the level of endothelial impairment. 
